# Tuning the Cell-Free Protein Synthesis System for Biomanufacturing of Monomeric Human Filaggrin

**DOI:** 10.3389/fbioe.2020.590341

**Published:** 2020-10-29

**Authors:** Jeehye Kim, Caroline E. Copeland, Kosuke Seki, Bastian Vögeli, Yong-Chan Kwon

**Affiliations:** ^1^Department of Biological and Agricultural Engineering, Louisiana State University, Baton Rouge, LA, United States; ^2^Department of Chemical and Biological Engineering, Northwestern University, Evanston, IL, United States; ^3^Chemistry of Life Processes Institute, Northwestern University, Evanston, IL, United States; ^4^Louisiana State University Agricultural Center, Baton Rouge, LA, United States

**Keywords:** cell-free synthetic biology, cell-free protein synthesis, filaggrin, protein therapeutics, bacteria codon bias, *Escherichia coli* cell lysate

## Abstract

The modern cell-free protein synthesis (CFPS) system is expanding the opportunity of cell-free biomanufacturing as a versatile platform for synthesizing various therapeutic proteins. However, synthesizing human protein in the bacterial CFPS system remains challenging due to the low expression level, protein misfolding, inactivity, and more. These challenges limit the use of a bacterial CFPS system for human therapeutic protein synthesis. In this study, we demonstrated the improved performance of a customized CFPS platform for human therapeutic protein production by investigating the factors that limit cell-free transcription–translation. The improvement of the CFPS platform has been made in three ways. First, the cell extract was prepared from the rare tRNA expressed host strain, and CFPS was performed with a codon-optimized gene for *Escherichia coli* codon usage bias. The soluble protein yield was 15.2 times greater with the rare tRNA overexpressing host strain as cell extract and codon-optimized gene in the CFPS system. Next, we identify and prioritize the critical biomanufacturing factors for highly active crude cell lysate for human protein synthesis. Lastly, we engineer the CFPS reaction conditions to enhance protein yield. In this model, the therapeutic protein filaggrin expression was significantly improved by up to 23-fold, presenting 28 ± 5 μM of soluble protein yield. The customized CFPS system for filaggrin biomanufacturing described here demonstrates the potential of the CFPS system to be adapted for studying therapeutic proteins.

## Introduction

Owing to the open nature of the cell-free biology, the cell-free protein synthesis (CFPS) system provides practicable opportunities to design and re-design the cellular processes outside the cell (Silverman et al., 2019). In the past decades, the CFPS system has received the spotlight as a versatile multipurpose toolkit of synthetic biology research because the core machinery of transcription and translation can be precisely tuned in an open environment of the *in vitro* platform, which is hard to achieve in the living system (Lu, 2017). The distinctive perspective of the CFPS system brings the technological advantages over traditional fermentation methods such as decoupling of protein synthesis from cell growth and reproduction, which allows engineers to solely utilize the catalyst from cells in an *in vitro* platform (Perez et al., 2016) and in artificial cell-like systems (Stano, 2019), enabling the study of protein synthesis process by examining the role of the supplements such as chaperones, elongation factors, ribosomes in a cell-free system (Li et al., 2014), and changing a paradigm of traditional logistic and storage practice by transforming the wet system to the dry format (Pardee et al., 2016; Stark et al., 2019; Wilding et al., 2019). In addition, continued efforts to develop a scalable CFPS system accelerate technology transfer and industrial adaptation (Zawada et al., 2011; Yin et al., 2012). Hence, the CFPS system takes its strong position in the race for the next-generation biomanufacturing platform as well as opens a new avenue for the precise synthesis of protein therapeutics.

Since protein therapeutics was first introduced a few decades ago, it possesses a substantial position in a modern pharmacotherapy market (Ram et al., 2016). The eukaryotic cell system has been widely accepted for protein therapeutics production because of supporting various post-translational modifications (Sanchez-Garcia et al., 2016). In contrast, the bacterial system, which has about 30% of biopharmaceuticals production, has not been considered a practical platform due to the lack of inherent modification capability (Chen, 2012). However, recent advances in the bacterial CFPS systems have overcome this technological barrier and equipped the synthetic capability of the protein therapeutics. i) The modern CFPS system has opened the opportunity of cell-free biomanufacturing of vaccines and personalized protein therapeutics by enabling cell-free protein glycosylation (Guarino and DeLisa, 2012; Jaroentomeechai et al., 2018; Kightlinger et al., 2018, 2020). ii) The synthesis of membrane proteins, which typically are a target of many drugs in clinical studies, has also been improved by the addition of membrane mimics such as peptide surfactants (Wang et al., 2011), membrane fragments (Shinoda et al., 2016), and liposomes (Matthies et al., 2011). iii) The on-demand vaccine productions have been achieved in the CFPS system as well. The CFPS platform was used in a portable medicines-on-demand system for a glycosylated protein vaccine (Adiga et al., 2018) and bioconjugate vaccine products (Stark et al., 2019). iv) The development and production of antibodies and antibody fragments by utilizing the CFPS system are available (Yin et al., 2012). High-throughput expression of IgG has achieved by disulfide bond isomerase (DsbC) addition, adjustment of redox potential (oxidized and reduced glutathione), chaperone and cofactor addition, and the tuning of the incubation time (Murakami et al., 2019). Also, the cytosol-penetrating antibodies have been synthesized in the CFPS system (Min et al., 2016). v) There have also been improvements in the CFPS systems devoted to the cancer therapeutic proteins such as onconase (Salehi et al., 2016) and crisantaspase (Hunt et al., 2019), demonstrating the CFPS platform to be advanced and on-demand technology for future cancer therapeutics.

Filaggrin (FLG) is a filament associated protein monomer that plays a crucial role in keratinization and maintaining the skin barrier function. The reduction of FLG in human epidermal skin increases the risk for asthma, immune sensitization, and other severe skin disorders such as atopic dermatitis and ichthyosis vulgaris (Cabanillas and Novak, 2016). In the human epidermis, the monomeric FLG is processed by FLG proteolysis from a large precursor profilaggrin. The skin aspartic protease (SASPase) is expressed exclusively in the stratum granulosum layer and cleaves the linker sequence of profilaggrin, leading to profilaggrin-to-FLG processing (Matsui et al., 2011). The free monomeric FLG protein is then cross-linked to keratin filaments by transglutaminases to form intracellular filaments that contribute to the compaction and mechanical strength of the cells (Cabanillas and Novak, 2016) and subsequently deiminated by peptidylarginine deiminases (PADs) 1 and 3 (O’Regan et al., 2008). During the deamination (namely, citrullination), the arginine residue in FLG is converted into citrulline, which drives a drastic charge loss and FLG dissociation from keratin filaments to further metabolism (Nachat et al., 2005). In the outer layers of the stratum corneum (SC), FLG monomers are degraded by proteases such as caspase-14, calpain-1, and bleomycin hydrolase into free amino acids (Egawa and Kabashima, 2018). These FLG breakdown products constitute the natural moisturizing factor (NMF) with the function of hydration or UV protection (Thyssen and Kezic, 2014).

The cell-free synthesized monomeric FLG can be used to restore impaired skin barrier function. There have been studies associated with FLG in regulating skin disorders by using gene therapy and protein therapeutics, including gene therapy targeting the upregulation of the FLG expression in the human immortalized keratinocyte cell line and mice for remedying atopic dermatitis (Otsuka et al., 2014). Another therapeutics study is a direct protein supplement. This approach required the precision delivery of monomeric FLG protein across the SC barrier, so that the reduced size of the FLG monomer was preferentially used rather than large profilaggrin for the therapy (Stout et al., 2014). Although the study by Stout et al. has shown the FLG synthesis using *Escherichia coli* host cell, the problem of lower production yield remains to be overcome to move on to future biomanufacturing of protein therapeutics. The overall protein synthesis rate can be improved by using an alternative *in vitro* biomanufacturing strategy with systemic optimization to set up the optimal production condition for the target protein of interest. Another perspective about FLG that makes this work meaningful, other than considering it just as a treatment of skin disorder, is that FLG is one of the many types of tandem repeat proteins that have a greater chance of mutation, which can render them dysfunctional. Due to their repetitive manner, the tandem repeats have higher mutation rates relative to other genomic loci, which contribute to generate variations that differ in the number of repeated units (Verstrepen et al., 2005). As the variable numbers of tandem repeats (VNTRs) mediate phenotypic plasticity and various polygenic disorders, a better understanding of tandem repeat polymorphisms (TRPs) and disease-associated mutations involving repeat instability needs to be accurately studied (Hannan, 2018). For example, there are three most common-sized human FLG genes encoding 10, 11, or 12 FLG tandem repeats due to the intragenic copy number variation in the gene (Brown and McLean, 2012). Synthesizing FLG repeat variants along with loss-of-function mutations and documenting the heterogeneity of amino acids in repeats will provide an insight on tandem repeat instability and disease-associated mutation. Many tandem repeats (including FLG) are present in coding and regulatory regions of the human genomes, and the mutations in the repeated units are often associated with many genetic diseases (Duitama et al., 2014). Besides, repetitive DNA sequences are common, consisting of almost half of the human genome (Jelinek et al., 1980). Hence, synthesizing and characterizing monomeric FLG will provide a better understanding of repeat variants associated genetics and pathogenesis of a range of human genetic diseases.

Here, in this study, we demonstrated that high-yield FLG synthesis (1.0 ± 0.2 mg/ml or equivalently 28 ± 6 μM) by streamlining the CFPS optimization process. First, we have chosen the eighth repeat unit of human profilaggrin (GenBank ID 2312) for CFPS as the monomeric FLG repeat does not possess a loss-of-function mutation site in the map of the profilaggrin gene reported previously, allowing a starting point to be set to study the variations of FLG subunit heterogeneity and mutations (Sandilands et al., 2007). The coding sequence [wild type (WT)] was then modified for the *E. coli* codon preference (codon optimization). The WT DNA fragment and *E. coli* codon-optimized DNA fragment were assembled into two separate plasmid backbones. Next, highly active crude cell extracts were prepared from BL21 Star (DE3) and Rosetta-gami B (DE3) pLacI *E. coli* strains to attain the highest FLG production rate. Finally, we assessed the CFPS reaction conditions not only for the synthesis of FLG but also for overlooking the potential for cell-free biomanufacturing of therapeutic proteins.

## Materials and Methods

### Plasmids and Strains

The bacterial plasmid pETBlue-1 (Novagen, St. Louis, MO) and pJL1-sfGFP were used for protein expression vectors. *Escherichia coli* strain Subcloning Efficiency^TM^ DH5α [Genotype F^–^ Φ80*lac*ZΔM15 Δ(*lac*ZYA-*arg*F) U169 *rec*A1 *end*A1 *hsd*R17(r_K_^–^, m_K_^+^) *pho*A *sup*E44 *thi*-1 *gyr*A96 *rel*A1 λ-] (Invitrogen, Waltham, MA) was used for subcloning. *E. coli* strains BL21 Star (DE3) [Genotype F^–^ *ompT hsdS*_B_ (r_B_^–^ m_B_^–^) *gal dcm rne131* (DE3)] (Invitrogen, Waltham, MA) and Rosetta-gami B (DE3) pLacI [Genotype F^–^ *ompT hsdS*_B_ (r_B_^–^ m_B_^–^) *gal dcm lacY1 ahpC* (DE3) *gor522*:Tn*10 trxB* pLacIRARE (Cam^R^, Kan^R^, Tet^R^)] (Novagen, St. Louis, MO) were used for protein expression.

### Growth Media

*E. coli* cells were grown in Luria–Bertani (LB) media or 2xYTPG media (16 g/L of tryptone, 10 g/L of yeast extract, 5 g/L of sodium chloride, 7 g/L of potassium phosphate dibasic, 3 g/L of potassium phosphate monobasic, pH 7.2, and 0.1 M of glucose) for cell biomass and protein expression.

### Preparation of Expression Vector

**FIGURE 1:**
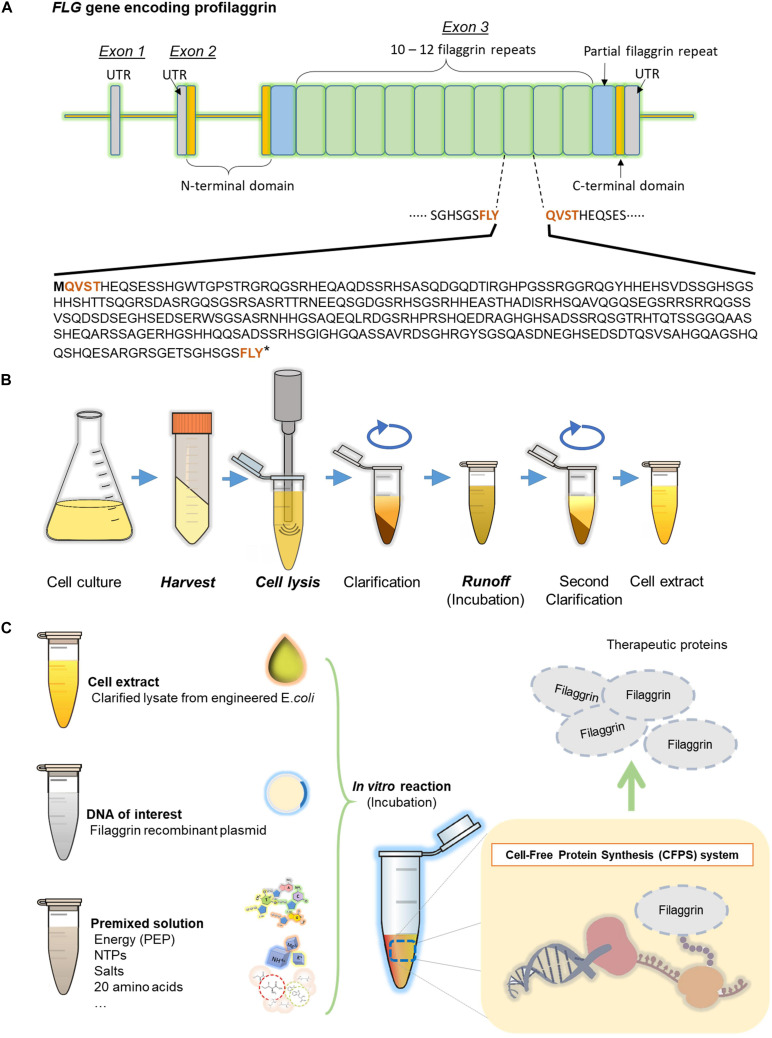
Filaggrin (FLG) gene to protein. **(A)** Human filaggrin tandem repeat sequences in the profilaggrin gene. The gene comprises three exons and two introns. Functional filaggrin repeat units (324 amino acids long, 10 to 12 repeats) are encoded in exon 3. The cleavage site (FLYQVST) is indicated in bold brown. **(B)** Cell extract preparation procedure. **(C)** The cell-free protein synthesis system.

The human eighth FLG repeat unit (324 amino acids) was selected for this study (Figure 1A). The WT nucleotide sequence was optimized for *E. coli* codon usage bias (strain K12) using a codon optimization tool (Integrated DNA Technologies) (Supplementary Figure 1). The WT and *E. coli* codon-optimized FLG coding sequences (so-called “Codon Opt” further on) were synthesized (Integrated DNA Technologies) and cloned into plasmids pJL1 and pETBlue-1 by Gibson Assembly (Gibson et al., 2009) for CFPS and *in vivo* protein synthesis, respectively. 6xhistidine tag was added to the C-terminal end of FLG during PCR. *E. coli* DH5α competent cells were used for the cloning host. The sequences have confirmed by DNA Sanger-Sequencing using the 3130xl Genetic Analyzer (Applied Biosystems). The recombinant plasmids were isolated by plasmid maxiprep kit (Invitrogen, Waltham, MA). A schematic cloning workflow is described in the Supplementary Figure 2.

### Preparation of Cell Extracts

#### Cell Culture and Harvest

*E. coli* cell extract was prepared as described previously (Figure 1B; Kwon and Jewett, 2015; Kim et al., 2019). *E. coli* BL21 Star (DE3) and Rosett-gami B (DE3) pLacI were grown in 2.5-L baffled Tunair shake flasks (IBI Scientific, Peosta, IA) at 37°C with vigorous shaking at 250 rpm. LB and 2xYTPG media were used for overnight seed culture (20 ml) and main culture (1 L), respectively. Cell cultures were monitored by measuring optical density at wavelength 600 nm (OD_600_) by UV-Vis spectrophotometer (Genesys 6, Thermo Fisher Scientific, Waltham, WA). The cultured cells were induced by 1 mM of isopropyl β-D-1-thiogalactopyranoside (IPTG) at OD_600_ 0.6 to regulate T7 RNA polymerase. The cultured cells were collected two times by centrifugation [5,000 relative centrifugal force (RCF) at 4°C for 15 min] at different phases: 0.5 L of cells was harvested at the exponential growth phase (OD_600_ 2.0), and the remaining cells were grown until they reached the stationary growth phase (OD_600_ 2.8–3.0) and harvested. Cell pellets were washed three times in Buffer A, weighed, flash-frozen in liquid nitrogen, and stored at −80°C freezer until use.

#### Cell Lysis, Runoff, and Cell Extract Clarification

The cell pellets were thawed on ice and resuspended in 1.3 ml of Buffer A per 1 g of wet cell mass. The resuspended cells were then transferred to clean microtube in ice for sonication (Qsonica Q125, Newtown, CT). The sonicator (model Q125) was set at 20-kHz frequency and 50% amplitude with 1/8” diameter probe. To lyse the cells, the pulse was on for 10 s and off for 10 s to minimize overheat protein degradation. Total energy input (joules) was varied in three levels (270, 537, and 1,074 J for 1 ml of cell suspension); 3 μl of 1 M dithiothreitol (DTT) was added to 1 ml of cell lysate, and the microtube was gently inverted several times. The cell lysates were then centrifuged at 12,000 RCF 4°C for 10 min. The supernatant (clarified cell lysate) was transferred to new microtubes. To compare the extract performance between non-runoff reaction and runoff reaction, half of the clarified cell lysate (cell extract) was aliquoted in a small volume (non-runoff reaction), and the second half was incubated at 37°C for 1 h (runoff reaction) and clarified by centrifugation (12,000 RCF at 4°C for 10 min). The supernatant was transferred to new tubes and aliquoted in a small volume. The aliquoted cell extracts were flash-frozen in liquid nitrogen and stored at −80°C freezer until use.

### Cell-Free Protein Synthesis

The CFPS was carried out for FLG expression as represented in Figure 1C. The standard 15 μl of CFPS reaction contains 4 μl of cell extract in addition to 12 mM of magnesium glutamate; 10 mM of ammonium glutamate; 130 mM of potassium glutamate; 1.2 mM of ATP; 0.85 mM each of GTP, UTP, and CTP; 34 μg/ml of L-5-formyl-5,6,7,8-tetrahydrofolic acid (folinic acid); 170 μg/ml of *E. coli* total tRNA, 57 mM of HEPES buffer (pH 7.2); 0.4 mM of nicotinamide adenine dinucleotide (NAD); 0.27 mM of coenzyme A; 4 mM of sodium oxalate; 1 mM of putrescine; 1.5 mM of spermidine; 2 mM of each of 20 amino acids; 33 mM of phosphoenolpyruvate (PEP); and 13.3 μg/ml of DNA template (plasmid). For the radioactivity measurement of cell-free synthesized protein, 10 μM of L-[U-^14^C]-leucine [300 mCi (11.1 GBq)/mmole, GE Life Sciences, Marlborough, MA] was added to the standard CFPS reaction. *E. coli* cell extract contains endogenous T7 RNA polymerase, which was synthesized from the IPTG-induced cells. The CFPS reactions were carried out at 30°C for 20 h.

### *In vivo* Protein Expression

The recombinant plasmid pJL1-FLG^His^ was transformed into Rosetta-gami B (DE3) pLacI [reactive oxygen species (ROS)] for *in vivo* FLG expression study (ROS:pJL1-FLG^His^). WT ROS and ROS:pJL1-FLG^His^ were grown in two 300-ml Tunair flasks with 100 ml of 2xYTPG media at 37°C with the vigorous shaking at 250 rpm. ROS was induced by 1 mM of IPTG when the cells’ optical density reached at 0.8 to inactivate lac repressor. Uninduced cell culture was prepared separately. The cultured cells were harvested by centrifugation. In order to lyse the cells, resuspended cells were disrupted by sonication (energy input 537 J for 1 ml of resuspended cells). The lysate was centrifuged at 14,000 RCF for 10 min to isolate a soluble fraction. The total cell protein and a soluble fraction were mixed with sample loading dye and denaturing solution (DTT), denatured by heating at 80°C for 5 min, and stored at −20°C until sodium dodecyl sulfate–polyacrylamide gel electrophoresis (SDS-PAGE) analysis.

### Protein Analysis

#### Trichloroacetic Acid-Insoluble Radioactivity Calculation

The amounts of cell-free synthesized proteins were quantified by measuring trichloroacetic acid (TCA)-insoluble radioactivity using a liquid scintillation counter (MicroBeta2, PerkinElmer, Waltham, MA). Radioactive L-[U-^14^C]-leucine added to the CFPS reaction mixture along with the 20 amino acids and carried out the standard CFPS reaction at 30°C for 20 h. The CFPS reaction was quenched by adding an equal volume of 0.5 M of potassium hydroxide and incubated at 37°C for 20 min. The same amounts of samples were spotted on two separate fiberglass paper sheets (Filtermat A, PerkinElmer, Waltham, MA) and dried at room temperature for 30 min. One of the fiberglass paper sheets was subjected to washing three times with 5% TCA solution at 4°C for 10 min. The sheet was rinsed in 100% ethanol at room temperature for 10 min to remove non-precipitated samples and was dried at room temperature for 40 min. The meltable cocktail (MeltiLex A, PerkinElmer, Waltham, MA) was heated to 95°C, and then radioactivity was measured using the liquid scintillation counter. The total and soluble yields were determined as described previously (Swartz et al., 2004).

#### Fluorescence Intensity and Quantification

The fluorescence intensity of active sfGFP was measured by multi-well plate fluorometer (Synergy HTX, BioTek, Winooski, VT); 2 μl of cell-free synthesized sfGFP was diluted with 48 μl of diethylpyrocarbonate (DEPC)-treated water (Invitrogen, Waltham, MA) in a 96-well half-area black flat-bottom plate (Corning, Glendale, AZ). The excitation and emission wavelengths for the sfGFP fluorescence were 485 and 528 nm, respectively. The cell-free synthesized sfGFP was purified by affinity chromatography cartridge (Strep-Tactin resin) following the manufacturer’s instruction (Qiagen, Germantown, MD). The soluble fraction of sfGFP was filtered by using a 0.2-μm syringe filter to remove remaining precipitants before passing through the column. Lysis buffer (50 mM of sodium phosphate monobasic, 300 mM of sodium chloride, pH 8.0) and elution buffer (50 mM of sodium phosphate monobasic, 300 mM of sodium chloride, 2.5 mM of desthiobiotin, and protease inhibitor) were filtered before use. The column was then washed three times with lysis buffer. Elution fractions were collected in the fresh tubes and concentrated first two fractions using an Amicon Ultra centrifugal filter unit [molecular weight cutoff (MWCO) (10 kDa)] (Millipore, Burlington, MA). The conversion factor was then calculated by Bradford assay using the purified sfGFP. The concentration of the sfGFP (μM) was determined by applying the conversion factor to the fluorescence intensity of the cell-free synthesized sfGFP.

#### Protein Electrophoresis

Five microliters of cell-free synthesized protein was mixed with 5 μl of sample buffer [NuPAGE LDS (4×), Invitrogen, Waltham, MA] and 10 μl of 200 mM of DTT and then mixed well. The samples were denatured by incubation at 80°C for 5 min before loading; 10 μl of heat-denatured protein samples was loaded on pre-casted gradient gel (NuPage 4-12% Bis-Tris Protein Gel, Invitrogen, Waltham, MA). The protein electrophoresis was carried out at 150 V for 75 min, and the proteins were visualized by Coomassie blue staining.

#### Filaggrin Purification

The cell-free synthesized FLG was isolated by immobilized-metal affinity chromatography (IMAC). The nitrilotriacetic acid (NTA) agarose matrices (Qiagen, Germantown, MD) were charged with Ni^2+^ and then washed with deionized water (Direct-Q system, Millipore, Burlington, MA) 10 times. All buffers contain the same composition of 50 mM of monosodium phosphate and 300 mM of sodium chloride with the different imidazole concentrations for each step of purification (10 mM for binding, 20 mM for wash, and 250 mM for elution). The CFPS reaction was transferred to a centrifugal filter unit [Amicon Ultra, MWCO (10 kDa)] and centrifuged at 14,000 × *g* RCF for 15 min. The filtrate was discarded, and an equal volume of lysis buffer containing protease inhibitors (Thermo Fisher, Waltham, MA) was added to the concentrate and subjected to repeated centrifuge to remove salts and other components in CFPS that possibly interfere with the histidine binding. The tagged proteins were then loaded onto the Ni-NTA matrices and immobilized, washed twice, and eluted by adding an elution buffer containing protease inhibitors. The imidazole in elute was removed by buffer exchange to Tris buffer (pH 8.0) containing protease inhibitors. The purified FLG was stored at −20°C until use.

### Statistical Analysis

All effect estimation was presented at the 5% significance level. Analyses were conducted using Graphpad Prism 8.4.3 (GraphPad Software). For the parametric analysis of data from quantification of the synthesized protein, two-way ANOVA followed by the Dunnett test was used.

## Results and Discussion

### Host Selection and Codon Optimization

#### Improving Protein Synthesis Using Rare tRNA Expressed Cell Extract

Since the transcription and translation apparatus is well conserved in the cell extract, host strain selection can revamp the overall protein synthesis rate in the CFPS system and likewise in *in vivo* recombinant protein synthesis platforms. Although the genetic code is universally shared across species, different codon usage biases strongly correlate with protein synthesis between the host strain and the origin of a gene of interest (Gingold and Pilpel, 2011). This codon usage impacts protein expression levels and folding efficiency during the translation (Quax et al., 2015). As the abundance of tRNA at the cellular level speeds up ribosomes to decode a codon (Novoa and de Pouplana, 2012), codon optimization and rare tRNA supplementation can improve transcription and translation efficiency without a shortage of tRNA availability. Therefore, choosing an appropriate *Escherichia coli* host that supports sufficient rare tRNA expression provides a decisive improvement in protein production yields, particularly in a prokaryotic heterologous gene expression system for a codon biased exotic gene such as FLG.

**FIGURE 2:**
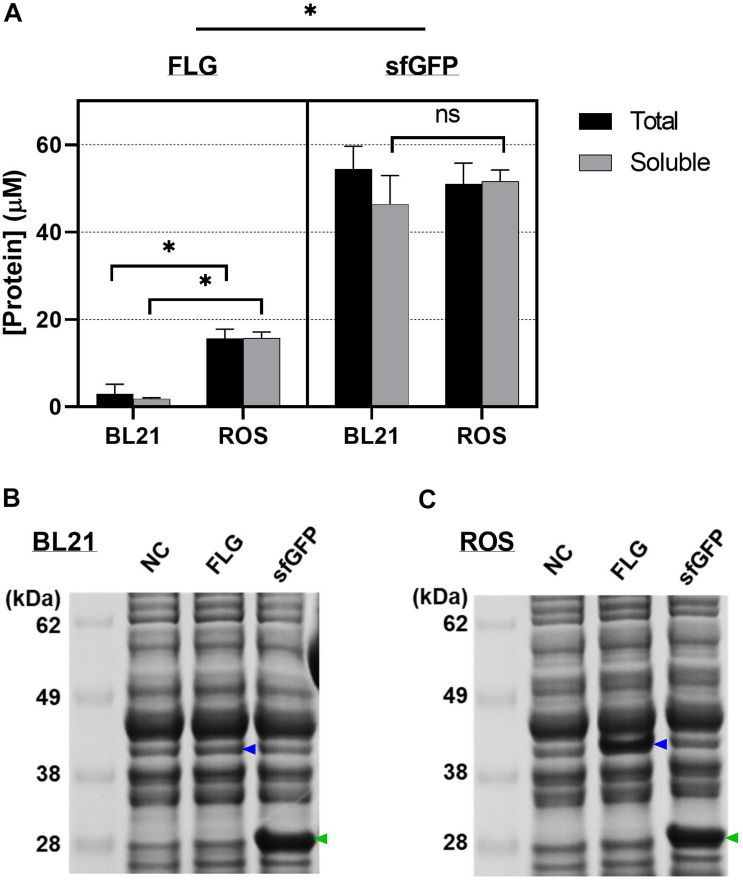
Cell-free protein synthesis (CFPS) using cell extracts from different *Escherichia coli* strains. **(A)** The amount of the cell-free synthesized protein was quantified by radioisotope analysis. sfGFP was used for the protein synthesis indicator (positive control). Data represented as the mean ± SD (*N* = 3). Tukey’s multiple comparisons test was performed with the two-way ANOVA. * indicates that the difference exists between the extracts from BL21 and reactive oxygen species (ROS) in terms of protein productivity (*p* < 0.001); ns indicates no significant difference between the two groups at the 0.05 level of significance. **(B)** The soluble portion of cell-free synthesized proteins using BL21 Star (DE3) cell extract. **(C)** The soluble portion of cell-free synthesized proteins using Rosetta-gami B (DE3) pLacI cell extract. NC represents a negative control that used no plasmid for the CFPS reaction. Filaggrin (FLG) and sfGFP represent that pJL1-FLG wild-type plasmid and pJL1-sfGFP plasmid were used for the CFPS reaction, respectively. The blue and green arrows indicate the cell-free synthesized FLG and sfGFP. All CFPS reactions were performed at 30°C for 20 h.

We first selected two *E. coli* strains BL21 Star (DE3) and Rosetta-gami B (DE3) pLacI (denoted as “BL21” and “ROS”) as hosts to validate and compare initial FLG synthesis level. ROS is a BL21 strain derivative. It is more versatile in producing eukaryotic proteins, including therapeutic proteins, as it supports six-rare-tRNA translation as well as disulfide bond formation. Both *E. coli* strains were cultured and lysed into cell extracts for the CFPS reactions. The separate reactions were conducted at 30°C for 20 h using the cell extracts from BL21 and ROS. The CFPS reaction with ROS cell extract showed a significantly higher yield than the synthesis using BL21 (*p* < 0.001) in both total and soluble fractions (increased yields by 5.3-fold and 9.1-fold, respectively) (Figure 2A). There were no significant differences between the BL21 and ROS extracts for sfGFP synthesis, indicating that two *E. coli* extracts are comparable for producing non-codon biased genes such as sfGFP. The SDS-PAGE results confirmed an increase in FLG production (blue arrows) using the ROS cell extract (Figures 2B,C). These results indicate that the additional supplement of rare tRNA in the CFPS reaction can improve the production yield of human proteins, which are considered one category of the hard-to-express proteins due to the highly biased codon usage. In other words, the gene encoding a FLG monomer (a functional repeat unit) contains 59 biased codons of its total 324 codons, which are not favorable in *E. coli* translational machinery due to lacking tRNA corresponding to the six rarely used codons in *E. coli* such as AGG, AGA for arginine, GGA for glycine, AUA for isoleucine, CUA for leucine, and CCC for proline. In this work, ROS cell extracts supplied the rare tRNAs to the CFPS system by utilizing the pLacIRARE plasmid containing a set of rare tRNA genes.

Another implication of the lower FLG monomer synthesis result here is that the expression level of the FLG monomer is more likely dependent on the CFPS reaction condition compared with the non-human-originated gene. Then, the protein synthesis yields would be improved by further optimizing the CFPS reaction condition to recreate a favorable environment for FLG monomer synthesis. On the basis of the assumption, we next modified the major contributors of the CFPS system systematically, as follows: DNA template, cell extract, and the CFPS reaction mixture.

#### Bypassing the Codon Usage via Codon Optimization

Codon optimization is an alternative method for addressing the biased codon usage in the CFPS system. When the canonical 20 amino acids are encoded within one to six synonymous codons, the frequencies of different codons vary across organisms (Gustafsson et al., 2004). Codon usage has been recognized as one of the most vital factors to regulate gene expression in the prokaryotic system (Lithwick and Margalit, 2003). Therefore, the complete reassignment strategy of human gene sequences for matching *E. coli* codon usage facilitates to maximize the chance of efficient protein expression.

**FIGURE 3:**
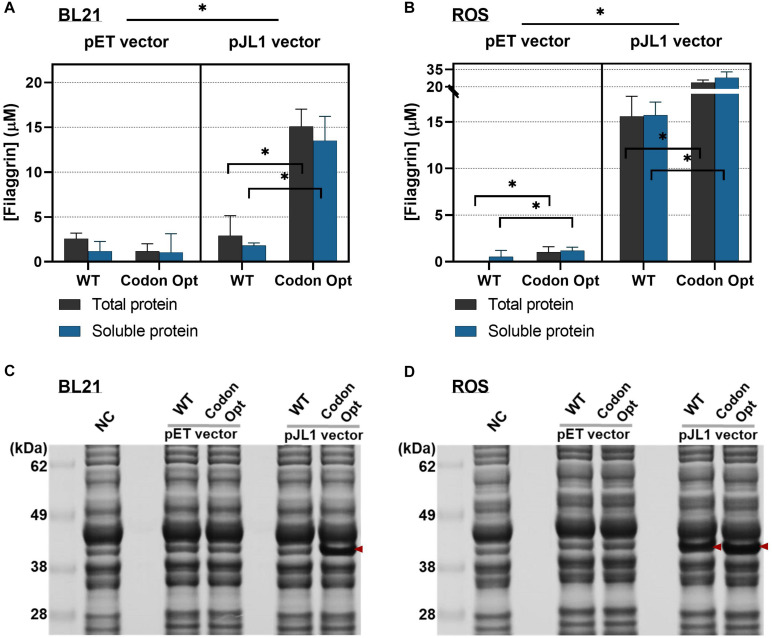
Cell-free synthesized protein yields from different filaggrin (FLG) expression vectors in the cell-free protein synthesis (CFPS). **(A)** FLG synthesis in BL21 extract. From the left to right, pETBlue-1-FLG WT, pETBlue1-FLG Codon Opt, pJL1-FLG WT, and pJL1-FLG Codon Opt. **(B)** FLG synthesis in reactive oxygen species (ROS) extract. From the left to the right, pETBlue-1-FLG WT, pETBlue1-FLG Codon Opt, pJL1-FLG WT, and pJL1-FLG Codon Opt. Data represented as the mean ± SD (*N* = 3). Tukey’s multiple comparisons test was used for *post hoc* analysis with two-way ANOVA. *p < 0.001. **(C)** and **(D)** Sodium dodecyl sulfate–polyacrylamide gel electrophoresis (SDS-PAGE) images of cell-free synthesized proteins with BL21 and ROS cell extract. The arrows indicate synthesized FLG. All CFPS reactions were performed at 30°C for 20 h.

To demonstrate this assumption, we synthesized the WT and codon-optimized FLG using IDT’s Codon Optimization Tool (organism set for *E. coli* K12). DNA properties, in particular, guanine–cytosine (GC) contents (%), were not changed significantly (WT, 57.03%; and codon optimized, 58.21%). We then cloned both sequences into pJL1 and pETBlue1 vectors to obtain four recombinant plasmids (pJL1-FLG WT, pJL1-FLG Codon Opt, pETBlue1-FLG WT, and pETBlue1-Codon Opt) by Gibson Assembly. The coding sequences were placed between the T7 promoter and T7 terminator in the plasmid. sfGFP sequence in the plasmid pJL1-sfGFP was substituted to the FLG sequence. pETBlue-1 vector is a compatible expression vector for the *E. coli* host strain Rosetta-gami B (DE3) pLacI. The yields of cell-free synthesized proteins were determined by radioisotope analysis and compared head to head (Figure 3).

Coupled with BL21 cell extract, the codon-optimized gene in pJL1 vector (pJL1-FLG Codon Opt) notably improved protein expression level than the WT gene (pJL1-FLG WT) in both total and soluble fractions (increased yields by 5.2-fold and 7.4-fold, respectively) (Figure 3A) (^∗^*p* < 0.001). In contrast, the codon-optimized gene in the pET vector did not show improved protein expression. Interestingly, pJL1-FLG Codon Opt paired with ROS cell extract increased protein yield only by 1.5-fold (total protein) and 1.8-fold (soluble protein) than did pJL1-FLG WT (Figure 3B) (^∗^*p* < 0.001). Regarding the low expression level shown with the pET vector, extra investigation showed that IPTG (0 to 1.0 mM) during the CFPS reaction could be supplemented. We expected efficient transcription initiation on the T7lac promoter located upstream of the FLG gene by IPTG inactivation of the lac repressor. However, the protein expression was still at a low level from uninduced (0 mM) to fully induced (1.0 mM) IPTG level (Supplementary Figure 3), implying that T7 RNA polymerase was not the limiting factor in the translation. The cell-free synthesized proteins visualized on SDS-PAGE indicate comparable results with those of the quantitative protein analysis (Figures 3C,D). Although the enhancement effect of codon optimization was lower when coupled with ROS extract (1.8-fold, Figure 3B, right panel) than with BL21 extract (7.4-fold, Figure 3A, right panel), the absolute protein yield increment was 12.2 μM (soluble protein) using ROS extract. This is not a negligible increase considering the entire production yield from pJL1-FLG Codon Opt paired with BL21 extract, which was 13.5 ± 2.7 μM. Conclusively, the codon optimization markedly enhanced FLG production in both BL21 and ROS cell extract-based CFPS system. Furthermore, the rare tRNA supplement by ROS extract combined with the *E. coli* codon-optimized gene has shown the combinational effect of the overall FLG synthesis yield up to 27.9 ± 5.0 (μM) in the soluble fraction (equivalently, 961 ± 172 μg/ml). This is 15.2-fold greater than that of BL21 extract combined with the WT gene. This result clearly indicated the synergic effect of changes in the plasmid construct and host strain.

### Tuning the Cell-Free Protein Synthesis System for Filaggrin Synthesis

#### Cell Extract Optimization

Multiple factors were explored in this study for preparing cell extract to improve FLG synthesis in the CFPS system. First, we tuned the cell culture and the time for cell harvest. Harvesting the cultured cells at the exponential phase of growth is beneficial for the performance of cell extracts because such cell extracts contain more active ribosomes and other translational machinery than other growth phases (Swartz, 2018). The time for harvest cells at high growth rate (OD_600_ = 2.0) was set to optimal harvest time (Harvest Opt), while the time for harvest cells at the stationary phase (2 h after reaching to maximum OD_600_ = 2.6∼3.0) was set to late harvest time (Harvest Late). Second, the level of cell lysis energy input (joules) is critical to generating highly active cell extract for the CFPS (Kwon and Jewett, 2015; Kim et al., 2019). Insufficient sonication energy lowers the total *E. coli* protein mass, which includes the translation apparatus and brings a higher chance of contamination by residual *E. coli* cells in the cell extract, while excessive energy input causes overheating degradation and deactivation of the proteins and enzymes in the cell extract (Kwon and Jewett, 2015). We tested the three different levels of energy input values representing insufficient energy (Low), optimal energy (Opt), and excessive energy (High). The optimal lysis energy was set to 537 J per processing volume (ml) for BL21 and ROS cell lysis; 269 and 1,074 J was used as Low and High lysis energy, respectively. Lastly, the runoff reaction was chosen as a critical post-lysis processing factor. Runoff reaction is a post-incubation procedure after cell lysis for decoupling ribosomal subunits from the paused translation on the endogenous mRNAs (polysome). In our previous work (Kim et al., 2019), the significant increase in protein yield was observed in the CFPS when using the cell extract prepared with runoff reaction. Dialysis was excluded in this study because we observed a decreased protein yield with dialyzed cell extract previously (Kim et al., 2019).

**FIGURE 4:**
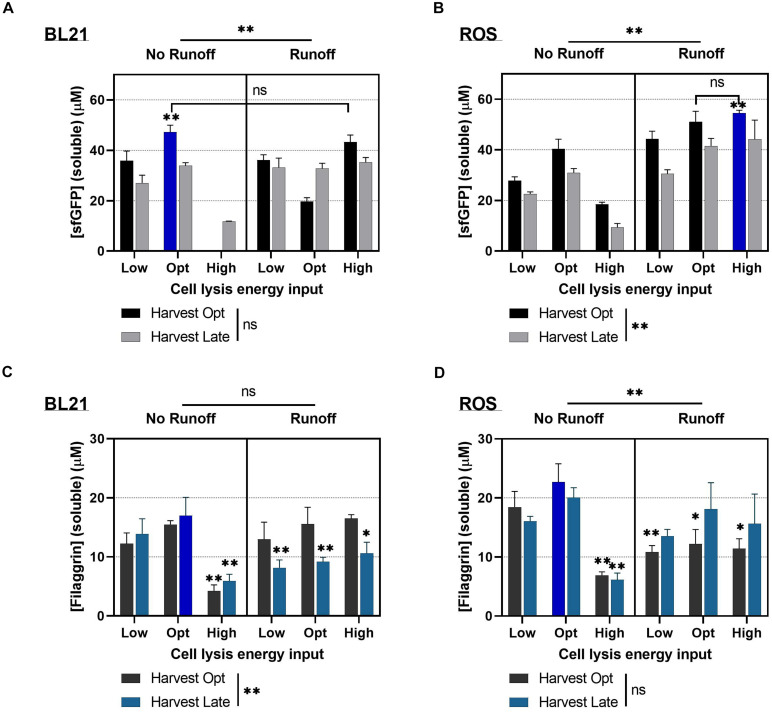
The effect of the combinatorial cell extract processing optimization for the cell-free protein synthesis (CFPS). A set of 12 different cell extracts was prepared for each strain by varying processing conditions with three nominal variables (harvest time, cell lysis energy input, and runoff). **(A)** The impact of processing conditions for BL21 Star (DE3) cell extract (BL21) for the sfGFP expression. **(B)** The impact of processing conditions for Rosetta-gami B (DE3) pLacI [reactive oxygen species (ROS)] cell extract for sfGFP expression. **(C)** The impact of processing conditions for BL21 cell extract for filaggrin (FLG) expression. **(D)** The impact of processing conditions for ROS cell extract for FLG expression. Data represented as the mean ± SD (*N* = 3). Followed by three-way ANOVA along with normality test, the multiple comparison test was performed for *post hoc* analysis. The Dunnett multiple comparisons were used by comparing the mean of a group showing the highest expression with the other group means (11 comparisons). *p < 0.05 and **p < 0.001 were presented when the difference exists between the control group and another group. All CFPS reactions were performed at 30°C for 20 h.

The combinatorial effect of the three critical cell extract processing factors on cell extract performance was studied by examining the protein yield of the 12 cell extract variations at four different scenarios: sfGFP expression using BL21 extracts (Figure 4A), sfGFP expression using ROS extracts (Figure 4B), FLG expression using BL21 extracts (Figure 4C), and FLG expression using ROS extracts (Figure 4D) (note that blue bars in each scenario indicate the optimal processing condition giving the highest protein yield). First, we compared the cell-free synthesized sfGFP expression levels using BL21/ROS extracts. As we expected, in Figure 4A, the sfGFP synthesis between standard CFPS reaction condition (Harvest Opt:Lysis Opt:No Runoff) and non-runoff condition (Harvest Opt:Lysis Opt:Runoff) in BL21 extract showed the same pattern reported previously (Kwon and Jewett, 2015). However, all the ROS extracts with runoff reactions showed improved sfGFP synthesis levels compared with those in non-runoff conditions (Figure 4B, right panel). Next, we studied the cell-free FLG synthesis in BL21/ROS extracts. The FLG synthesis in BL21-based CFPS has shown the highest yield in the combinatorial condition of Harvest Late:Lysis Opt:No Runoff in Figure 4C (note that this optimal processing condition significantly improved FLG expression compared with that in the groups in ^∗^ and ^∗∗^). The highest FLG yields for ROS-based CFPS were obtained in a combinational condition of Harvest Opt:Lysis Opt:No Runoff (Figure 4D).

Interestingly, the runoff reaction was able to help to regain the protein synthesis capacity of the cell extract processed with excessive energy (Figure 4A, both panels, right bars). The increased pattern was observed in all scenarios (Figure 4, Harvest Opt or Late:Lysis High:Runoff) as well. This result indicated the runoff reaction could be an essential step for generating cell extracts that show homogeneous protein synthesis capacity batch by batch. We assume that the excessive sonication condition provided sufficient energy to rupture the most *E. coli* cell wall and release the cytoplasmic components, maximizing the level of endogenous mRNA, genomic DNA, other small molecules, and cell debris that potentially interferes the exogenous gene expression during the CFPS. The runoff reaction can assist in degrading such molecules, which results in reactivating translational machinery in the cell extract processed by the excessive sonication. On the other hand, without the runoff reaction, optimal sonication energy input is considered the major affecter to gain the highest extract performance (Figure 4, left panels, middle bars). The overall performance of the CFPS system in terms of protein expression was shown to be significantly affected by cell extract processing conditions. With the combinatorial processing condition for the cell extract, the soluble protein yield in the CFPS increased up to 54 ± 1 μM (1,463 ± 32 μg/ml) for the sfGFP and 23 ± 3 μM (799 ± 109 μg/ml). Total FLG proteins showed the same expression pattern as the soluble portion (Supplementary Figure 4).

Even though the cell extract from processing combination of Harvest Opt:Lysis Opt:No Runoff provided a high protein yield in general, there were slight variations in optimal processing condition, which depends on the type of proteins as well as the type of expression hosts. Thus, it is necessary to customize the processing condition to obtain the cell extract specialized for a certain protein cell-free biomanufacturing.

#### Modifying Cell-Free Protein Synthesis Reaction Conditions

The optimal CFPS conditions vary from protein to protein because of the inherent complexity of protein, gene transcription and translation, and post-translational modification. In the CFPS system, the gene transcription and translation that initiated by the addition of DNA templates are processed simultaneously in both prokaryotic and eukaryotic systems (Zemella et al., 2015). Modifying the CFPS conditions favorable to the specific target protein synthesis can increase the system’s protein synthesis capability. The incubation temperature is the important factor of protein translation dynamics and directly influences the speed of the transcription and translation-elongation rate, protein folding, and solubility (Yu et al., 2015). Recent researches have shown the synonymous codon substitution affects the translation-elongation rate as well (Spencer et al., 2012; Supek et al., 2014). In addition, magnesium ions (Mg^2+^) play a critical role in protein synthesis. Lacking or having a shortage of magnesium ions causes incorrect folding of ribosomal RNA and disassembly of the ribosome, which results in early translation termination (Pontes et al., 2016). In this work, we assessed the effect of incubation temperature and the concentration of magnesium ions to explore the optimal condition for FLG synthesis during the CFPS reaction.

**FIGURE 5:**
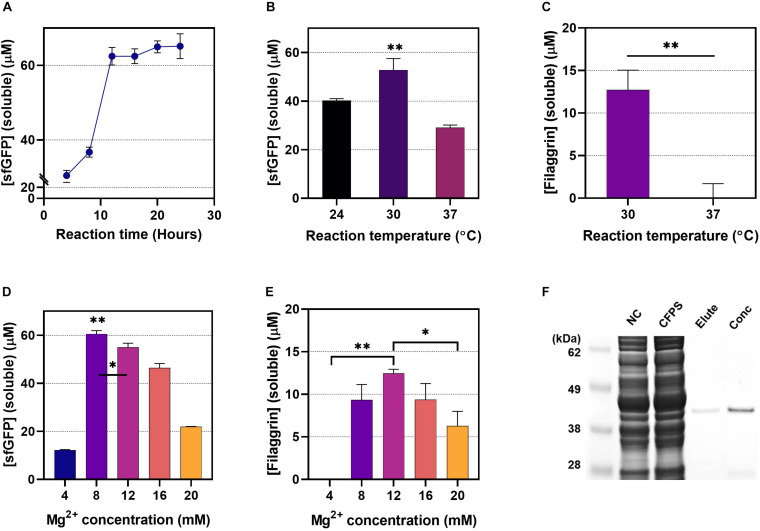
The effect of the cell-free protein synthesis (CFPS) reaction conditions. **(A)** 24-h monitoring of sfGFP expression (CFPS reaction: 30°C, 8 mM of Mg^2+^). **(B)** The incubation temperature for sfGFP expression. The CFPS reaction was carried out at different temperatures (24°C, 30°C, and 37°C) for 20 h using 12 mM of Mg^2+^. **(C)** The incubation temperature for filaggrin (FLG) expression. The CFPS reaction was carried out at different temperatures (30°C and 37°C) for 20 h using 12 mM of Mg^2+^. **(D)** The effect of the concentrations of Mg^2+^ for sfGFP synthesis (CFPS reaction: 30°C, 20 h). **(E)** The effect of the concentrations of Mg^2+^ for FLG synthesis (CFPS reaction: 30°C, 20 h). Data represented as the mean ± SD (*N* = 3). The unpaired t-test and the one-way ANOVA with the Dunnett test were performed. The Dunnett multiple comparisons were used by comparing the mean of a group showing the highest expression with the other group means *p < 0.05, **p < 0.001. **(F)** Gel electrophoresis of cell-free synthesized and purified FLG. Lane 1, protein size marker; lane 2, CFPS without FLG plasmid; lane 3, soluble FLG in CFPS; lane 4, isolated FLG protein (elute from Ni-NTA IMAC); lane 5, concentrated FLG. The pJL1-FLG^His^ plasmid was used as the expression vector in the reactive oxygen species (ROS) cell extract-based CFPS system.

The sfGFP expression level was measured at 4-h intervals to gauge the appropriateness of the 20 h of incubation to set as default reaction time when evaluating the effect of the CFPS conditions on protein yield (Figure 5A). The sfGFP synthesis increased quickly for the first 10 h of reaction, and then the curve plateaued after 12–16 h of incubation, implying that 20 h is sufficient CFPS reaction time to complete the CFPS reaction at maximum yield when comparing yields at different CFPS conditions. Since we observed that the soluble proteins are comparable with the total protein level in FLG and sfGFP expression (Figure 2A), we measured the soluble protein in different temperatures [24°C, 30°C (default), and 37°C] to find out the optimal CFPS reaction. The CFPS reaction was set for 20 h. Both sfGFP and FLG showed the highest soluble protein yield in the 30°C reaction (Figures 5B,C). Besides that, the CFPS at 30°C produces a highly soluble form of FLG, which was observed little in the CFPS at 37°C (Supplementary Figure 5A), implying that a reaction at 30°C helps the proper protein folding in the *E. coli* CFPS system. We assume that both the FLG gene’s codon optimization and the temperature alteration increased the overall protein folding dynamics, particularly the translation-elongation rate of the FLG synthesis. For example, unlike the FLG expression in human, during the *E. coli* CFPS at 37°C, the T7 RNA polymerase-mediated transcription rate and *E. coli* translation-elongation rate would be too fast to have time for protein folding. By lowering the temperature to 30°C, transcription and translation rate would slow down, which provides time for proper protein folding. The optimal concentration of magnesium ion in the CFPS was determined to be 8 mM for sfGFP expression (Figure 5D), which was the consistent outcome reported by Dudley et al. (2020). However, the FLG expression was at the highest level in the CFPS system when 12 mM of Mg^2+^ concentration was used (Figure 5E and Supplementary Figures 5B,C). With the optimized CFPS reaction condition, we successfully isolated and concentrated the cell-free synthesized FLG observed to have a clean single band without noise (Figure 5F).

### Filaggrin Expression *in vivo*

After achieving a high-yield FLG synthesis via optimizing the CFPS system in this study, we next explore the FLG synthesis *in vivo*. The CFPS system has been compared with *in vivo* recombinant protein synthesis platform in terms of usability, flexibility, scalability, and, more importantly, productivity. One of the features is that the CFPS is accomplished in a single reaction, whereas the *in vivo* system requires multiple procedures for protein expression (Rosenblum and Cooperman, 2014). This allowed the CFPS to become an essential tool to rapidly screen and eliminate factors limiting the production of target proteins. For example, we identify that the codon bias was the main limiting factor in FLG expression without conducting time-consuming steps of cloning, transformation, and cell cultivation. This highlights the versatility of cell-free platform as a protein production optimization toolkit. Additionally, the CFPS system does not require host strain modification to accommodate the exogenous DNA to express target protein, which causes the poor growth of the host, protein inactivity, and low protein yields (Rosano and Ceccarelli, 2014). Not only the exogenous protein expression but also the presence of the exogenous gene in bacteria can be a burden to the host cell (Andersson et al., 1996).

**FIGURE 6:**
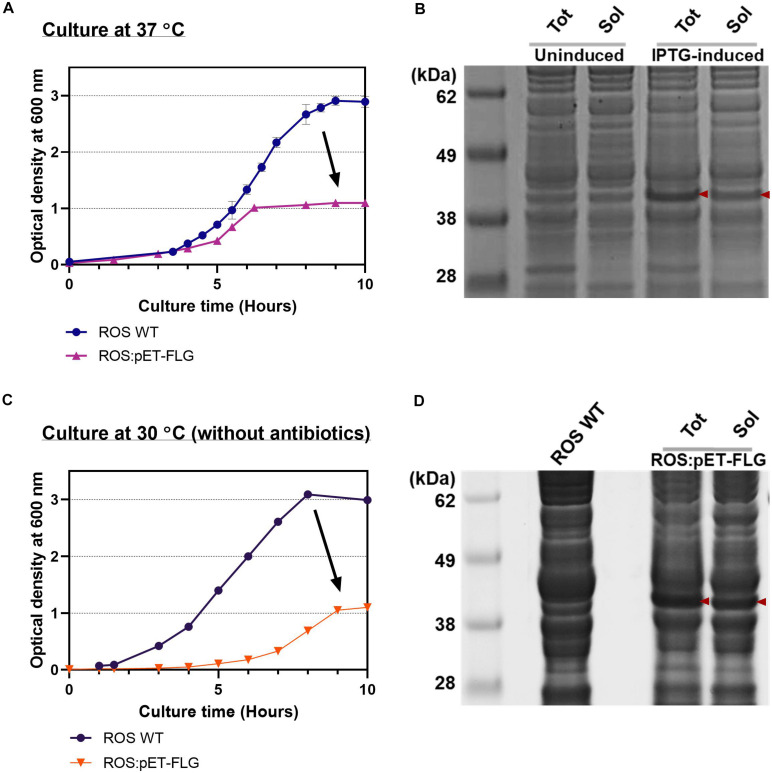
Filaggrin (FLG) expression *in vivo*. **(A)** The cell growth curve of reactive oxygen species (ROS) wild type (WT) and ROS:pET-FLG at 37°C with antibiotics. **(B)** Productivity and solubility of induced and non-induced FLG. **(C)** The cell growth curve of ROS WT and ROS:pET-FLG at 30°C without antibiotics. **(D)** Productivity and solubility of synthesized FLG. Red arrows indicate FLG. Tot, total proteins; Sol, soluble proteins.

As the FLG production yield greatly improved through the systemic approach with cell-free platform, the optimized condition was simply applied to the *in vivo* system to stress the suitability of cell-free system as a simulator of FLG production *in vivo*. The ROS cell extract showed promising performance to synthesize FLG *in vitro* due to the endogenous rare tRNA supplement from the plasmid pLacIRARE (Figure 3B). The ROS cell extract was prepared following the standard cell extract preparation protocol, as described in *Preparation of Cell Extracts*. We first examined *in vivo* protein synthesis to confirm the FLG synthesis at a higher temperature (37°C) by using the host strain with the plasmid pETBlue1-FLG^His^ [Rosetta-gami B (DE3):pETBlue1-FLG^His^ or simply ROS:pET-FLG]. We observed the slow growth of ROS:pET-FLG compared with WT ROS (ROS WT) (Figure 6A). The cell doubling time (T*_d_*) of ROS WT and ROS:pET-FLG was 46 and 51 min, respectively. IPTG induction was successfully regulated FLG gene translation compared with non-induced cells. However, ROS:pET-FLG cell growth reached at the stationary phase earlier, implying the presence of inhibiting effect. The same trend appeared in IPTG-induced (at OD_600_ = 0.6) and uninduced cell culture. This indicates that the presence of plasmid significantly inhibited cell growth and eventually achieved the lower FLG synthesis yield and solubility (Figure 6B). This inhibitory effect results in the difficulty of rapid optimization for target protein expression when using *in vivo* system.

Next, the cell culture condition was slightly modified, similar to the CFPS reaction condition, mainly by lowering culture temperature to 30°C to gain soluble protein synthesis. Also, cell culture, protein expression, and cell harvest conditions were slightly modified to mimic the CFPS condition, which was optimized in this study. For example, ROS WT and ROS:pET-FLG cells were grown in 2xYTPG media at 30°C without antibiotics, IPTG induction at OD_600_ = 0.6, and harvest the cell at OD_600_ = 2.0 (OD_600_ = 1.0 for ROS:pET-FLG) (Figure 6C); washed with Buffer A; and disrupted the cell via optimized sonication condition. As a result, we observed increased protein productivity (Figure 6D). However, the *in vivo* study revealed that the FLG expression causes a negative effect on cell growth and implies the possible low protein yield that resulted from the reduction of total cell biomass. Consequently, the CFPS with the processing optimization can detour this limitation to ensure the protein synthesis for future biomanufacturing of therapeutic proteins.

## Conclusion

Although the profit of therapeutic proteins in the pharmaceutics market is significantly expanded in the last few decades, the manufacturing capacity and production cost limit the market availability of biological drugs (Matthews et al., 2017). In this work, we proposed the CFPS system to streamline human therapeutic protein production. In this customized CFPS model, the therapeutic protein FLG expression was significantly improved, representing 28 ± 5 μM of soluble protein yield. As the most FLG protein was expressed with a soluble form in the CFPS system, the protein synthesis enhancement by optimization was evaluated using the amount soluble fraction of FLG. The protein yield was 9.1 times greater with the rare tRNA overexpressing host strain (ROS extract) than with the host strain without rare tRNA (BL21 extract) in the CFPS system. The combination of rare tRNA overexpressing host and DNA codon bias optimization enhanced protein synthesis by up to 15.2-fold of protein yield in the CFPS system. To improve the FLG production yield, we applied the combinational optimization of the three key manufacturing processes without the individual factor optimization. We obtained the 3.7-fold (ROS extract) and 4.0-fold (BL21 extract) improvement of protein yields in the customized CFPS system. Taken together, the customized CFPS system for FLG synthesis described here not only provides the alternative protein synthesis platform but also proves the potential of the CFPS system for biomanufacturing of human therapeutic proteins. In this study, we focused on the high-yield soluble FLG production using the CFPS. Solubility would not be the solid indicator for functionality, but it suggested the proper protein folding and an important prerequisite of it. The follow-up characterization and functionality studies will expand therapeutics applications and capabilities of *in vitro* protein synthesis.

## Data Availability Statement

The original contributions presented in the study are included in the article/Supplementary Material, further inquiries can be directed to the corresponding author.

## Author Contributions

Y-CK conceived and supervised the project. JK and Y-CK designed and conceptualized experiments. JK, CEC, KS, and BV performed the experiments and acquired the data. JK, CEC, KS, BV, and Y-CK analyzed data. JK wrote the original manuscript. JK, CEC, and Y-CK revised and edited the manuscript. All authors contributed to the article and approved the submitted version.

## Conflict of Interest

The authors declare that the research was conducted in the absence of any commercial or financial relationships that could be construed as a potential conflict of interest.
