# Supplementary material for: Tuning the Cell-Free Protein Synthesis System for Biomanufacturing of Monomeric Human Filaggrin
Source: Front Bioeng Biotechnol. 2020 Oct 29;8:590341. doi: 10.3389/fbioe.2020.590341 (PMC7658397; doi:10.3389/fbioe.2020.590341)
Supplement: Supplementary file 1 [file Data_Sheet_1.docx]

Supplementary Material

Tuning the cell-free protein synthesis system for biomanufacturing of monomeric human filaggrin

Jeehye Kim^1^, Caroline E. Copeland^1^, Kosuke Seki^2,3^, Bastian Vögeli^2^, and Yong-Chan Kwon^1,4,*^

^1^Department of Biological and Agricultural Engineering, Louisiana State University, Baton Rouge, LA 70803, USA

^2^Department of Chemical and Biological Engineering, Northwestern University, Evanston, IL 60208, USA

^3^Chemistry of Life Processes Institute, Northwestern University, Evanston, IL 60208, USA

^4^Louisiana State University Agricultural Center, Baton Rouge, LA 70803, USA

***Correspondence:**Yong-Chan Kwon
yckwon@lsu.edu

ATGCAAGTCAGCACACACGAGCAGTCGGAAAGTTCTCATGGTTGGACAGGTCCATCAACTCGTGGTCGCCAAGGATCACGTCACGAGCAAGCACAAGATTCTAGCCGCCATTCTGCGAGCCAGGATGGCCAGGATACGATTCGCGGGCATCCTGGTAGCTCGCGTGGTGGACGTCAAGGTTATCACCACGAGCACAGTGTTGATTCGTCGGGGCACTCAGGTTCGCACCATAGCCACACGACGTCACAGGGACGCTCGGACGCGAGCCGTGGACAGTCGGGGTCTCGTTCAGCATCACGTACAACTCGTAATGAAGAGCAATCTGGCGATGGCAGTCGTCATAGCGGCAGTCGCCACCATGAGGCCTCAACTCACGCAGACATCTCTCGCCATTCTCAAGCGGTTCAAGGACAATCGGAAGGTAGCCGCCGCAGTCGCCGCCAAGGATCTAGCGTTTCCCAGGACTCAGATTCAGAGGGACATTCAGAAGACTCCGAACGTTGGTCTGGTAGTGCGTCCCGCAATCACCATGGCTCCGCCCAGGAGCAACTTCGCGACGGGTCACGCCATCCACGCAGTCACCAGGAAGATCGCGCGGGTCATGGACATTCAGCGGATAGTTCTCGCCAGAGCGGGACTCGTCATACGCAGACCTCGTCGGGAGGTCAGGCAGCTTCAAGTCATGAGCAAGCACGTAGCAGTGCGGGAGAGCGTCACGGCTCGCATCATCAGCAGAGTGCAGACAGCTCTCGTCACTCGGGGATCGGGCATGGTCAAGCTAGTTCGGCTGTTCGTGATTCCGGGCATCGTGGGTACTCGGGCAGCCAAGCGTCGGACAACGAGGGACATTCGGAAGACAGTGATACACAGTCAGTGAGCGCGCACGGGCAGGCTGGTTCCCACCAACAATCGCACCAAGAGTCGGCTCGCGGGCGCTCTGGTGAAACAAGCGGACATTCGGGATCCTTTTTATACTAATAA

**Supplementary Figure 1.** Gene sequence of *E. coli* codon-optimized Filaggrin 8^th^ repeat (981 bp, including the start codon (ATG) and two stop codons (TAATAA)).


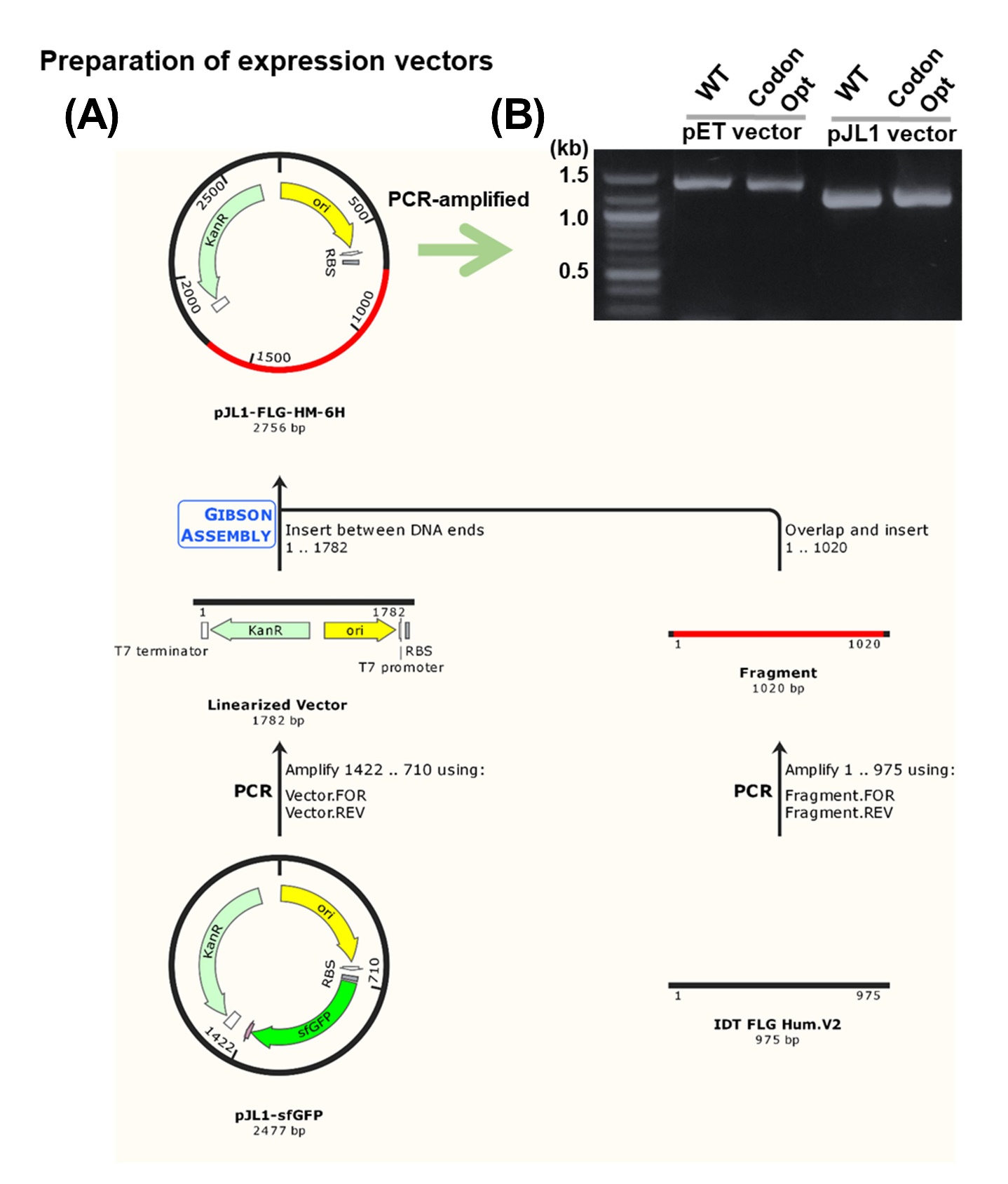
**Supplementary Figure 2.** **Preparation of expression vectors.** **(A)** a schematic cloning workflow for expression vectors preparation. **(B)** the PCR-amplified gene fragments from the expression vectors. The size of wild-type and codon-optimized FLG gene fragment is identical. The size of the PCR-amplified fragment, including the overlapping sequences with linearized vectors, vary. The gene sizes are 1389 bp from pETBlue-1 and 1174 bp for pJL1-based recombinant plasmids.

**
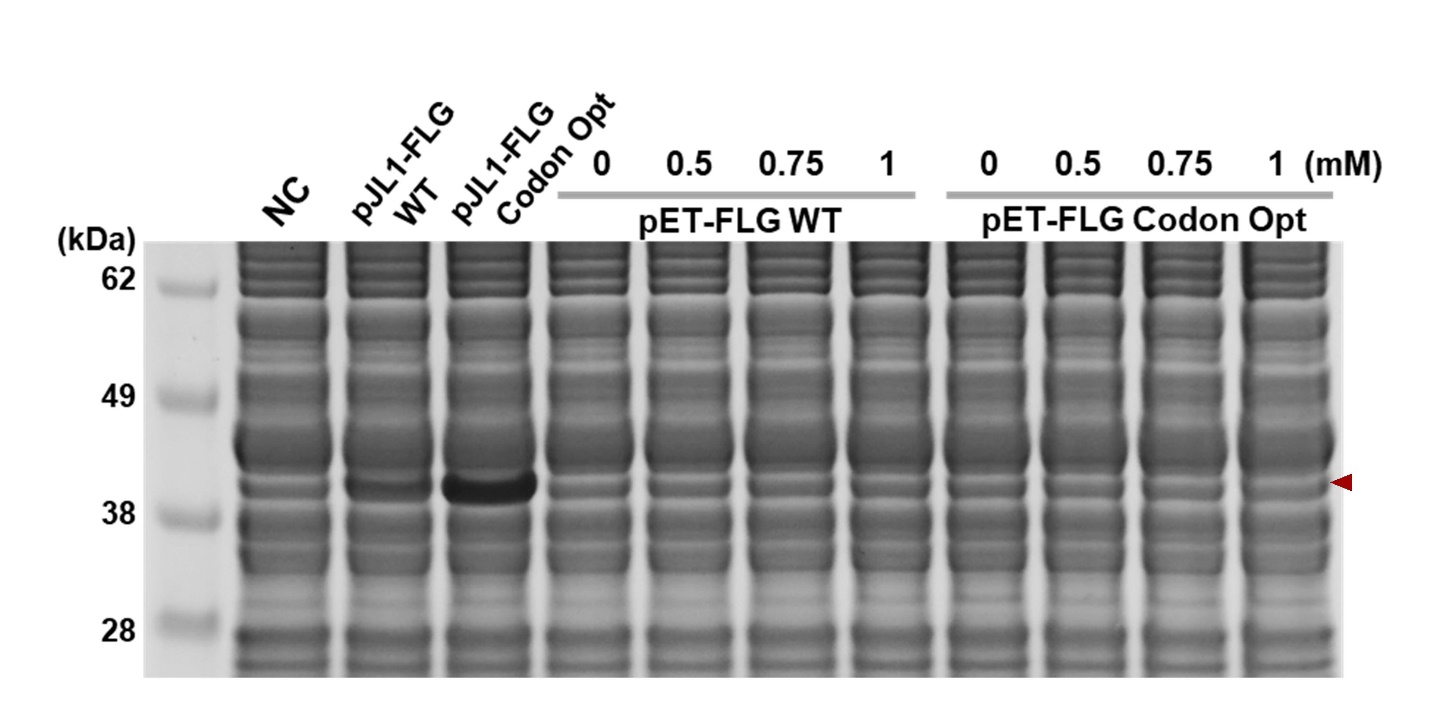
Supplementary Figure 3.** **The effect of the supplemental IPTG in the CFPS reaction (ROS extract).** The arrows indicate the synthesized FLG in the CFPS. Lane 1: protein size marker, lane 2: negative control without DNA, lane 3: pJL1-FLG WT addition, lane 4: pJL1-FLG Codon Opt addition, lane 5-8: pET-FLG WT addition (IPTG supplement concentration: 0, 0.5, 0.75, and 1 mM), lane 9-12: pET-FLG Codon Opt addition (IPTG supplement concentration: 0, 0.5, 0.75, and 1 mM). All CFPS reactions were performed at 30 °C for 20 hours.

**
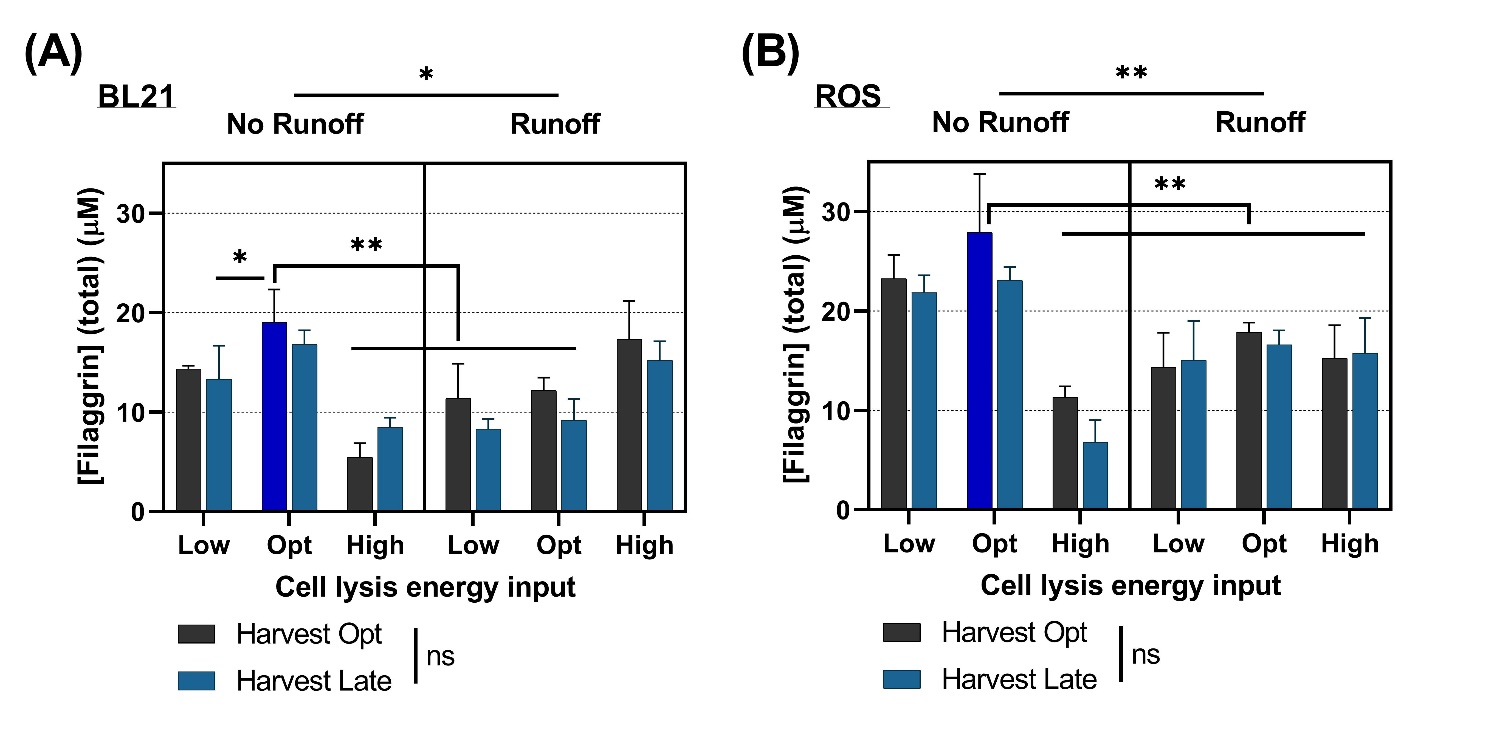
Supplementary Figure 4**. **The effect of the optimized cell-free extract for FLG expression. (A)** the FLG (total) protein yield using BL21 cell extract prepared in twelve processing variations. **(B)** the FLG (total) protein yield using ROS cell extract with twelve processing variations. Data represented as the mean ± SD (N = 3). Followed by three-way ANOVA along with normality test, the multiple comparison test was performed for Post hoc analysis. The Dunnett's multiple comparisons were used by comparing the mean of a group showing the highest expression with the other group means (11 comparisons). * *p* < 0.05, ** *p* < 0.001 was presented when the difference exists between the control group and another. All CFPS reactions were performed at 30 °C for 20 hours. The pJL1-FLG Codon Opt plasmid was used as the FLG expression vector for the BL21/ROS CFPS system.

**
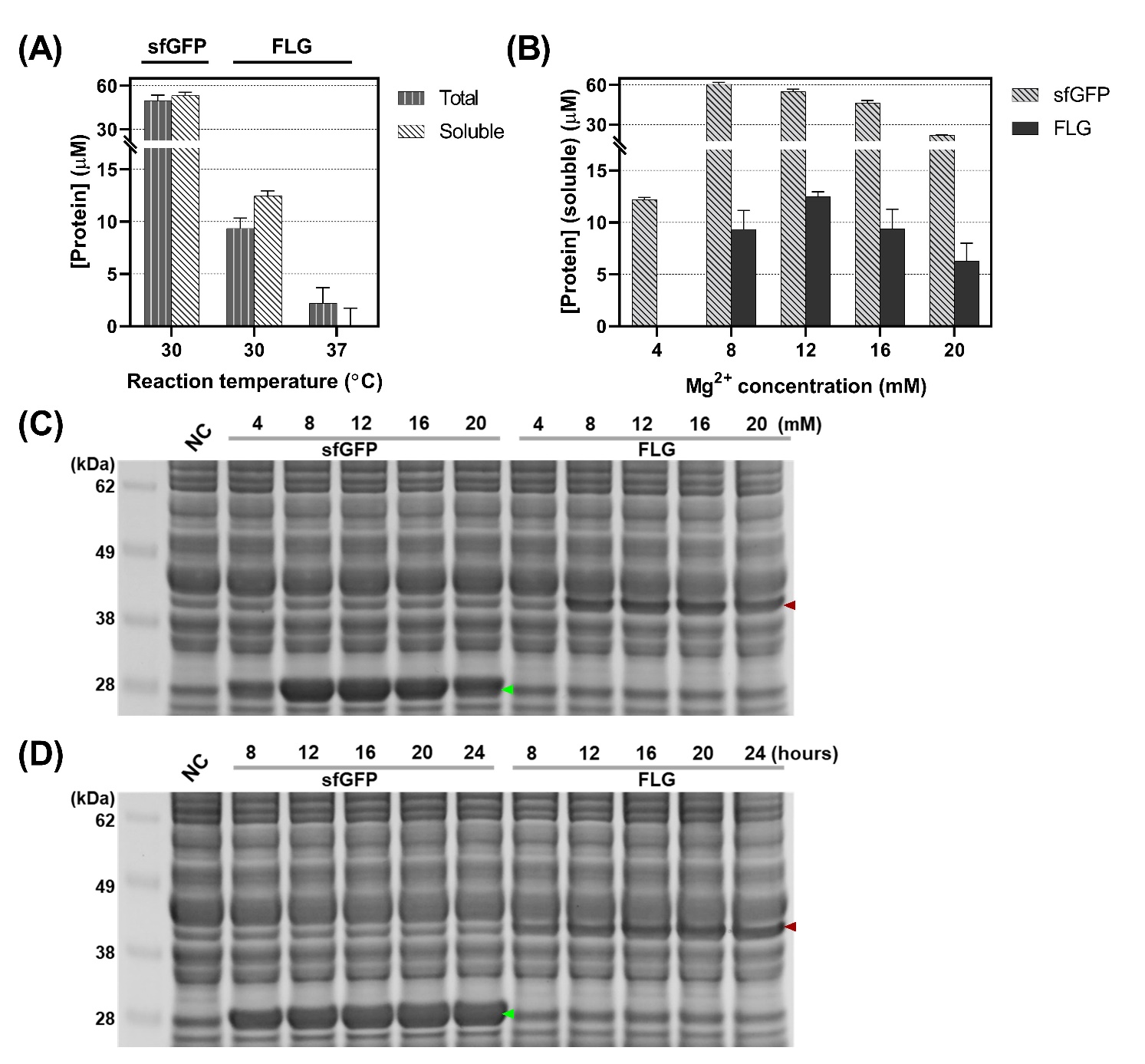
Supplementary Figure 5. Solubility of cell-free synthesized sfGFP and FLG, and optimization of Mg^2+^ and reaction time.** The green arrows indicate sfGFP and red arrows indicate FLG. **(A)** the sfGFP and FLG expression in CFPS at different incubation temperatures. **(B)** a side-by-side comparison of sfGFP and FLG expression at different Mg^2+^ concentrations. **(C)** the effect of magnesium ions concentration in CFPS. Lane 1: protein size marker, lane 2: negative control without DNA, lane 3-7: sfGFP expression at 4, 8, 12, 16, and 20 mM of Mg^2+^ concentration, lane 8-12: FLG expression at 4, 8, 12, 16, and 20 mM of Mg^2+^ concentration. **(D)** protein expression by incubation time. Lane 1: protein size marker, lane 2: negative control, lane 3-7: sfGFP expression at different reaction time (8, 12, 16, 20, and 24 hours), lane 8-12: FLG expression at reaction time (8, 12, 16, 20, and 24 hours).
